# Strong sum distance in fuzzy graphs

**DOI:** 10.1186/s40064-015-0935-5

**Published:** 2015-05-06

**Authors:** Mini Tom, Muraleedharan Shetty Sunitha

**Affiliations:** Department of Mathematics, SCMS School Of Engineering and Technology, Karukutty, 683 582 Kerala India; Department of Mathematics, National Institute of Technology Calicut, Kozhikode, 673 601 Kerala India

**Keywords:** Fuzzy graph, Strong sum distance, Fuzzy cycle, Fuzzy tree, Boundary, Interior

## Abstract

In this paper the idea of strong sum distance which is a metric, in a fuzzy graph is introduced. Based on this metric the concepts of eccentricity, radius, diameter, center and self centered fuzzy graphs are studied. Some properties of eccentric nodes, peripheral nodes and central nodes are obtained. A characterisation of self centered complete fuzzy graph is obtained and conditions under which a fuzzy cycle is self centered are established. We have proved that based on this metric, an eccentric node of a fuzzy tree *G* is a fuzzy end node of *G* and a node is an eccentric node of a fuzzy tree if and only if it is a peripheral node of *G* and the center of a fuzzy tree consists of either one or two neighboring nodes. The concepts of boundary nodes and interior nodes in a fuzzy graph based on strong sum distance are introduced. Some properties of boundary nodes, interior nodes and complete nodes are studied.

## Introduction

The theory of fuzzy graphs was developed by Rosenfeld (1975) in the year 1975. During the same time Yeh and Bang (1975) have also introduced various connectedness concepts in fuzzy graphs. Rosenfled (1975) introduced the concept of *μ*−distance in fuzzy graphs. The author has defined *μ*− length of any *u*−*v* path *P* as the sum of reciprocals of arc weights in *P* and distance between *u* and *v* called the *μ*−distance denoted by *d*_*μ*_(*u*,*v*), as the the smallest *μ*− length of *P*. In a fuzzy graph *G*:(*V*,*σ*,*μ*), *d*_*μ*_(*u*,*v*) is a metric on *V* ∀ *u*,*v*∈*V*. Based on this *μ*− distance Bhattacharya (1987) has introduced the concepts of eccentricity and center in fuzzy graphs and the properties of this metric are further studied by Sunitha and Vijyakumar (1998). The geodetic iteration number and geodetic number of fuzzy graphs based on *μ*−distance was introduced by Linda and Sunitha (2013). Abdul Jabbar et al. (2009) introduced the concept of fuzzy planar graph and discussed some of its interesting properties. Recently, Pal et al. (2013) and Samanta et al. (2014) introduced and investigated the concept of fuzzy planar graphs and studied several properties. Noura and Akram (2014) studied isomorphism between intuitionistic fuzzy planar graphs. Interval valued fuzzy planar graphs and interval valued fuzzy dual graph are defined by Tarasankar et al. (2014). Some properties of interval valued fuzzy planar graphs and interval valued fuzzy dual graph are also studied by the authors. Talebi and Rashmanlou (2013) studied isomorphism on interval valued fuzzy graph. Rashmanlou and Pal (2013) defined isometry on interval valued fuzzy graphs and established that isometry on interval valued fuzzy graphs is an equivalence relation. The same authors (Pal and Rashmanlou 2013) also defined irregular interval valued fuzzy graphs and their various classifications. Recently, Akram et al. introduced the concepts of bipolar fuzzy graphs and interval-valued fuzzy line graphs (Akram 2011, 2012, 2013; Akram and Dudek 2011, 2012). Further the author has defined length, distance, eccentricity, radius and diameter of a bipolar fuzzy graph and has introduced the concept of self centered bipolar fuzzy graphs (Akram and Karunambigai 2011). Namboothiri et al. (2013) discussed Cayley fuzzy graphs. Alshehri and Akram (2013) introduced the concept of Cayley bipolar fuzzy graphs and investigated some of their properties. The author has also introduced the concept of an antipodal intuitionistic fuzzy graph and self median intuitionistic fuzzy graph of the given intuitionistic fuzzy graph (Akram and Karunambigai 2012). Akram and Alshehri (2014) introduced various types of intuitionistic fuzzy bridges, intuitionistic fuzzy cut vertices, intuitionistic fuzzy cycles and intuitionistic fuzzy trees in intuitionistic fuzzy graphs and investigated some of their interesting properties. To model ecological problems, in 1968 Cohen (1968) introduced the notion of competition graphs. Fuzzy competition graph was introduced by Samanta and Pal (2013). Two generalizations of fuzzy competition graph as fuzzy *k*-competition graphs and *p*-competition fuzzy graphs are also defined by the same authors. In Samanta et al. (2015) define another generalization of fuzzy competition graph, called *m*-step competition graph. Bhutani and Rosenfeld have introduced the concepts of strong arcs (Bhutani and Rosenfeld 2003a), fuzzy end nodes (Bhutani and Rosenfeld 2003c) and *g*−distance in fuzzy graphs (Bhutani and Rosenfeld 2003b). The geodesic eccentricity and geodesic center of a fuzzy graph *G* is also discussed in (Bhutani and Rosenfeld 2003b). Further studies based on the *g*−distance are carried out by Sameena and Sunitha (2008) and (Sameena and Sunitha 2011). The concepts of *g*−peripheral nodes, *g*−boundary nodes and *g*−interior nodes based on *g*−distance was introduced by Linda and Sunitha (2012). Nagoorgani and Umamaheswari introduced the concept of fuzzy detour *μ*−distance (Nagoorgani and Umamaheswari 2010). The authors further defined fuzzy detour *μ*−center and studied its properties. Fuzzy detour *g*−distance was introduced by Linda and Sunitha (2014a) and in (Linda and Sunitha 2014b), the authors introduced fuzzy detour *g*− boundary nodes and fuzzy detour *g*− interior nodes in fuzzy graphs. In this paper we introduce the concept of strong sum distance in fuzzy graphs and a study on boundary nodes and interior nodes of a fuzzy graph based on this distance is carried out.

Section 2 contains preliminaries and in section 3, strong sum distance in fuzzy graphs is defined and proved that it is a metric. Based on this metric, eccentricity, radius, diameter, center in fuzzy graphs are defined. Necessary conditions for a fuzzy graph to be self centered are obtained in this section. By an example it is shown that a unique eccentric node fuzzy graph with each node eccentric need not be self centered. Sufficient conditions for a fuzzy cycle to be self centered is given in section 4. A necessary and sufficient condition for a complete fuzzy graph to be self centered is given in section 5. In section 6 we have the embedding theorem i.e; construction of fuzzy graph *G* from a given fuzzy graph *H* such that <*C*(*G*)> ≅*H*. In section 7, based on this metric it is proved that an eccentric node of a fuzzy tree *G* is a fuzzy end node of *G* and a node is an eccentric node of a fuzzy tree if and only if it is a peripheral node of *G* and the center of a fuzzy tree consists of either one or two neighboring nodes. In section 8 boundary node of a fuzzy graph based on strong sum distance is defined. Boundary nodes of fuzzy tree and complete fuzzy graph are discussed in this section. A complete node is defined and it is showed by an example that a complete node need not be a boundary node. Also an example to show that fuzzy cut node can be a boundary node is given in this section. Interior of a fuzzy graph based on strong sum distance is defined in Section 9. In a fuzzy graph there are nodes which are neither boundary nodes nor interior nodes. Interior node in complete fuzzy graph and boundary nodes in a cycle are also discussed in this section.

## Preliminaries

A fuzzy graph(f-graph) (Mordeson and Nair 2000) is a triplet *G*:(*V*,*σ*,*μ*) where *V* the vertex set, *σ* is a fuzzy subset of *V* and *μ* is a fuzzy relation on *σ* such that *μ*(*u*,*v*) ≤*σ*(*u*) ∧*σ*(*v*) ∀*u*,*v* ∈*V*. We assume that *V* is finite and non empty, *μ* is reflexive and symmetric. In all the examples *σ* is chosen suitably. Also we denote the underlying crisp graph (Harary 1969) by *G*^∗^ :(*σ*^∗^,*μ*^∗^) where *σ*^∗^ = {*u*∈*V* : *σ*(*u*) > 0 } and *μ*^∗^ = {(*u*,*v*) ∈*V* x *V* : *μ*(*u*,*v*) >0}. Here we assume *σ*^∗^=*V*. A fuzzy graph *H*:(*V*,*τ*,*ν*) is called a partial fuzzy subgraph of *G*:(*V*,*σ*,*μ*) if *τ*(*u*) ≤ *σ*(*u*) ∀*u* ∈*τ*^∗^ and *ν*(*u*,*v*) ≤ *μ*(*u*,*v*) ∀(*u*,*v*) ∈*ν*^∗^. In particular we call *H*:(*V*,*τ*,*ν*) a fuzzy subgraph of *G*:(*V*,*σ*,*μ*) if *τ*(*u*) = *σ*(*u*) ∀u ∈*τ*^∗^ and *ν*(*u*,*v*) = *μ*(*u*,*v*) ∀(*u*,*v*) ∈*ν*^∗^ and if in addition *τ*^∗^=*σ*^∗^, then *H* is called a spanning fuzzy subgraph of *G*. A weakest arc of *G*:(*V*,*σ*,*μ*) is an arc with least membership value. A path *P* of length *n* is a sequence of distinct nodes *u*_0_,*u*_1_,⋯,*u*_*n*_ such that *μ*(*u*_*i*−1_,*u*_*i*_) > 0, *i* = 1,2,3, ⋯,*n* and the degree of membership of a weakest arc in the path is defined as its strength. If *u*_0_=*u*_*n*_ and *n*≥ 3, then *P* is called a cycle and a cycle *P* is called a fuzzy cycle(f-cycle) if it contains more than one weakest arc. A fuzzy graph *G*:(*V*,*σ*,*μ*) is a complete fuzzy graph (CFG) if *μ*(*u*,*v*)=*σ*(*u*)∧*σ*(*v*),∀*u*,*v*∈*σ*^∗^.

The strength of connectedness between two nodes *u* and *v* is defined as the maximum of the strengths of all paths between *u* and *v* and is denoted by *CONN*_*G*_(*u*,*v*). A *u*−*v* path *P* is called a strongest *u*−*v* path if its strength equals CONN _*G*_(*u*,*v*). A fuzzy graph *G*:(*V*,*σ*,*μ*) is connected if for every *u*,*v* in *σ*^∗^, *CONN*_*G*_(*u*,*v*) > 0. Throughout this, we assume that *G* is connected. An arc of a fuzzy graph is called strong if its weight is at least as great as the strength of connectedness of its end nodes when it is deleted and a *u*−*v* path is called a strong path if it contains only strong arcs (Bhutani and Rosenfeld 2003a). If *μ*(*u*,*v*) > 0, then *u* and *v* are called neighbors. Also *v* is called a strong neighbor if arc (*u*,*v*) is strong. The set of all neighbors of *u* is denoted by *N*(*u*) and the set of all strong neighbors of *u* is denoted by *N*_*s*_(*u*). A node *u* is a fuzzy end node of *G* if it has exactly one strong neighbor in *G*. A strong path *P* from *u* to *v* is a *u*−*v* geodesic if there is no shorter strong path from *u* to *v* and the length of a *u*−*v* geodesic is the geodesic distance from *u* to *v* denoted by *d*_*g*_(*u*,*v*) (Bhutani and Rosenfeld 2003b). Consider the fuzzy graphs *G*_1_:(*V*_1_,*σ*_1_,*μ*_1_) and *G*_2_:(*V*_2_,*σ*_2_,*μ*_2_) with $\sigma _{1}^{*}$ = *V*_1_ and $\sigma _{2}^{*}$ = *V*_2_. An isomorphism (Bhutani 1989) between two fuzzy graphs *G*_1_ and *G*_2_ is a bijective map *h* : *V*_1_ → *V*_2_ that satisfies *σ*_1_(*u*) = *σ*_2_(*h*(*u*)) ∀*u* ∈ *V*_1_ and *μ*_1_(*u*,*v*) = *μ*_2_(*h*(*u*),*h*(*v*)) ∀ *u*,*v* ∈ *V*_1_ and is denoted by *G*_1_ ≅ *G*_2_.

An arc (*u*,*v*) is a fuzzy bridge(f-bridge) of *G* if deletion of (*u*,*v*) reduces the strength of connectedness between some pair of nodes (Rosenfeld 1975). Equivalently, (*u*,*v*) is a fuzzy bridge if and only if there exist *x*,*y* such that (*u*,*v*) is an arc on every strongest *x*−*y* path. A node is a fuzzy cutnode (f-cutnode) of *G* if removal of it reduces the strength of connectedness between some other pair of nodes (Rosenfeld 1975). Equivalently, *w* is a fuzzy cutnode if and only if there exist *u*,*v* distinct from *w* such that *w* is on every strongest *u*−*v* path. A connected fuzzy graph *G*:(*V*,*σ*,*μ*) is a fuzzy tree (f-tree) if it has a spanning fuzzy subgraph *F*:(*V*,*σ*,*ν*), which is a tree, where for all arcs (*u*,*v*) not in *F* there exists a path from *u* to *v* in *F* whose strength is more than *μ*(*u*,*v*). Thus for all arcs (*u*,*v*) which are not in *F*, *μ*(*u*,*v*)< *CONN*_*F*_(*u*,*v*). A maximum spanning tree (MST) of a connected fuzzy graph *G*:(*V*,*σ*,*μ*) is a fuzzy spanning subgraph *T*:(*V*,*σ*,*ν*) such that *T*^∗^ is a tree and for which $\sum \limits _{u \neq v }$*ν*(*u*,*v*) is maximum (Mordeson and Nair 2000). Note that for a fuzzy tree *G*, maximum spanning tree is unique and is the spanning fuzzy subgraph *F* itself (Sunitha and Vijayakumar 1999). Depending on the *CONN*_*G*_(*u*,*v*) of an arc (*u*,*v*) in a fuzzy graph *G*, strong arcs are further classified as *α*−strong and *β*−strong and the remaining arcs are termed as *δ*−arcs (Sunil and Sunitha 2009) as follows. Note that *G*−(*u*,*v*) denotes the fuzzy subgraph of *G* obtained by deleting the arc (*u*,*v*) from *G*. An arc (*u*,*v*) in *G* is called *α*−strong if *μ*(*u*,*v*) > *CONN*_*G*−(*u*,*v*)_(*u*,*v*). An arc (*u*,*v*) in *G* is called *β*−strong if *μ*(*u*,*v*) = *CONN*_*G*−(*u*,*v*)_(*u*,*v*). An arc (*u*,*v*) in *G* is called a *δ*−arc if *μ*(*u*,*v*)< *CONN*_*G*−(*u*,*v*)_(*u*,*v*). A *δ*−arc (*u*,*v*) is called a *δ*^∗^− arc if *μ*(*u*,*v*)> *μ*(*x*,*y*) where (*x*,*y*) is a weakest arc of *G*.

## Strong sum distance in fuzzy graph

Rosenfeld (1975) has defined *μ*− length of any *u*−*v* path *P* as the sum of reciprocals of arc weights in *P* and distance between *u* and *v* called the *μ*−distance denoted by *d*_*μ*_(*u*,*v*), as the the smallest *μ*− length of *P*. Here we introduce a new definition for length of any *u*−*v* path *P* in a fuzzy graph *G* and based on the new definition we introduce the concept of strong sum distance.

### **Definition3.1**.

Let *G*:(*V*,*σ*,*μ*) be a connected fuzzy graph. For any path *P*:*u*_0_−*u*_1_−*u*_2_−*u*_3_−⋯⋯−*u*_*n*_, length of *P* is defined as the sum of the weights of the arcs in *P* i.e. *L*(*P*) = $\displaystyle \sum \limits _{i=1}^{n} \,\mu (u_{i-1},u_{i})$. If *n* = 0, define *L*(*P*) = 0 and for *n*≥ 1, *L*(*P*)>0. Also if *G* is disconnected then *L*(*P*) may be zero. For any two nodes *u*,*v* in *G*, let **P**={*P*_*i*_:*P*_*i*_ is a strong *u*−*v* path,*i*=1,2,3,⋯ }. The strong sum distance between *u* and *v* is defined as *d*_*ss*_(*u*,*v*) = Min {*L*(*P*_*i*_):*P*_*i*_∈**P**,*i*=1,2,3,⋯ }.

### **Remark3.2**.

If *μ*(*u*,*v*) = 1 ∀ (*u*,*v*) ∈*μ*^∗^ then *d*_*ss*_(*u*,*v*) is the length of the shortest path as in crisp graph.

### **Theorem3.3**.

In a fuzzy graph *G*:(*V*,*σ*,*μ*), *d*_*ss*_ : *V* ×*V* → [0,1] is a metric on *V*. i.e. ∀ *u*,*v*,*w*∈*V*(1) *d*_*ss*_(*u*,*v*) ≥ 0 ∀ *u*,*v*∈*V*(2) *d*_*ss*_(*u*,*v*)= 0 if and only if *u*=*v*(3) *d*_*ss*_(*u*,*v*) = *d*_*ss*_(*v*,*u*)(4) *d*_*ss*_(*u*,*w*)≤ *d*_*ss*_(*u*,*v*)+*d*_*ss*_(*v*,*w*)

### **Proof**.

(1) and (2) follows from the definition. Next, since reversal of a strong path from *u* to *v* is a strong path from *v* to *u* and vice versa, *d*_*ss*_(*u*,*v*) = *d*_*ss*_(*v*,*u*). Let *P*_1_ be a strong *u*−*v* path such that *d*_*ss*_(*u*,*v*) = *L*(*P*_1_) and *P*_2_ be a strong *v*−*w* path such that *d*_*ss*_(*v*,*w*) = *L*(*P*_2_). The strong path *P*_1_ followed by strong path *P*_2_ is a *u*−*w* walk and since every walk contains one path, there exists a strong *u*−*w* path in *G* whose length is at most *d*_*ss*_(*u*,*v*) + *d*_*ss*_(*v*,*w*).Therefore, *d*_*ss*_(*u*,*w*) ≤ *d*_*ss*_(*u*,*v*) + *d*_*ss*_(*v*,*w*).

### **Definition3.4**.

Let *G*:(*V*,*σ*,*μ*) be a connected fuzzy graph and let *u* be a node of *G*. The eccentricity *e*(*u*) of *u* is the strong sum distance to a node farthest from *u*. Thus *e*(*u*) = max {*d*_*ss*_(*u*,*v*):*v* ∈*V*}. For a node *u*, each node at strong sum distance *e*(*u*) from *u* is an eccentric node for *u* denoted by *u*^∗^. *G* is a unique eccentric node (u.e.n) fuzzy graph if each node in *G* has a unique eccentric node. The radius *r*(*G*) is the minimum eccentricity of the nodes, whereas the diameter *d*(*G*) is the maximum eccentricity. A node *u* is a central node if *e*(*u*) = *r*(*G*), and *C*(*G*) is the set of all central nodes. The fuzzy subgraph induced by *C*(*G*) denoted by <*C*(*G*)> = *H*:(*V*,*τ*,*ν*) is called the center of *G*. A connected fuzzy graph G is self centered if each node is a central node i.e. *G*≅*H*. A node *u* is a peripheral node if *e*(*u*) = *d*(*G*).

### **Example3.5**.

**Figure 1:**
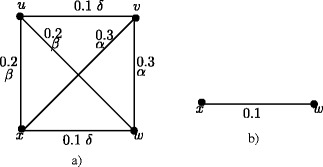
Nodes in fuzzy graph *G* based on strong sum distance.**(a)**. Eccentric nodes, Central nodes, Peripheral nodes **(b)**. Center of *G*.

In Figure 1, *d*_*ss*_(*u*,*v*) = 0.5, *d*_*ss*_(*u*,*w*) = 0.2, *d*_*ss*_(*u*,*x*) = 0.2, *d*_*ss*_(*v*,*w*) = 0.3, *d*_*ss*_(*v*,*x*) = 0.3, *d*_*ss*_(*w*,*x*) = 0.4. Therefore *e*(*u*) = 0.5, *u*^∗^ = *v*, *e*(*v*) = 0.5, *v*^∗^ = *u*, *e*(*w*) = 0.4, *w*^∗^ = *x*, *e*(*x*) = 0.4, *x*^∗^ = *w*. The central nodes are *w* and *x*. The peripheral nodes are *u* and *v*. Here *r*(*G*) = 0.4 and *d*(*G*) = 0.5.

### **Remark3.6**.

In crisp graph, a unique eccentric node graph is self centered if and only if each node of *G* is eccentric. Note that the f-graph in Figure 1 is a unique eccentric node f-graph. In Figure 1, each node is eccentric and each node has a unique eccentric node but *G* is not self centered.

### **Theorem3.7**.

For any connected fuzzy graph *G*:(*V*,*σ*,*μ*), the radius and diameter satisfy *r*(*G*) ≤ *d*(*G*) ≤ 2 *r*(*G*).

### **Proof**.

*r*(*G*) ≤ *d*(*G*) follows from the definition of radius and diameter. Let *w* be a central node of *G*. Therefore *e*(*w*) = *r*(*G*). Let *u* and *v* be two peripheral nodes of *G*. Therefore *e*(*u*) = *e*(*v*) = *d*(*G*).By triangle inequality *d*_*ss*_(*u*,*v*) ≤ *d*_*ss*_(*u*,*w*) + *d*_*ss*_(*w*,*v*)i.e. *d*(*G*) ≤ *r*(*G*) + *r*(*G*). *d*(*G*) ≤ 2 *r*(*G*). Therefore *r*(*G*) ≤ *d*(*G*) ≤ 2 *r*(*G*).

### **Remark3.8**.

**Figure 2:**
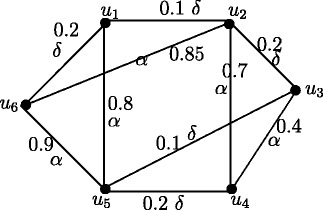
Fuzzy graph in which difference of eccentricities of the adjacent nodes is greater than 1.

Note that in crisp graph, eccentricities of the adjacent nodes differ atmost by 1. In Figure 2, *u*_1_ and *u*_2_ are adjacent nodes. Note that *e*(*u*_1_) = 3.65 and *e*(*u*_2_) = 2.55 and hence |*e*(*u*_1_)) - *e*(*u*_2_)| = 1.1. But this result is true if *u* and *v* are strong neighbors as in following theorem.

### **Theorem3.9**.

For every two strong neighbors *u* and *v* in a connected fuzzy graph *G*:(*V*,*σ*,*μ*), |*e*(*u*) - *e*(*v*)| ≤ 1.

### **Proof**.

Assume without loss of generality *e*(*u*) ≥ *e*(*v*). Let *x* be a node farthest from *u*. i.e. *e*(*u*) = *d*_*ss*_(*u*,*x*) ≤ *d*_*ss*_(*u*,*v*) + *d*_*ss*_(*v*,*x*), by triangle inequality. Therefore *e*(*u*) ≤ *d*_*ss*_(*u*,*v*) + *e*(*v*), since *e*(*v*) ≥ *d*_*ss*_(*v*,*x*). Since *u* and *v* are strong neighbors we have *d*_*ss*_(*u*,*v*) ≤ 1. Therefore *e*(*u*)≤ 1 + *e*(*v*) ⇒ 0 ≤ *e*(*u*) - *e*(*v*) ≤ 1.$\therefore $ |*e*(*u*) - *e*(*v*)| ≤ 1.

The above Theorem can be generalized as follows.

### **Theorem3.10**.

For every two nodes *u* and *v* in a connected fuzzy graph *G*:(*V*,*σ*,*μ*), |*e*(*u*) - *e*(*v*)| ≤ *d*_*ss*_(*u*,*v*).

### **Proof**.

Assume without loss of generality *e*(*u*) ≥ *e*(*v*). Let *x* be a node farthest from *u*. i.e. *e*(*u*) = *d*_*ss*_(*u*,*x*) ≤ *d*_*ss*_(*u*,*v*) + *d*_*ss*_(*v*,*x*), by triangle inequality. Therefore *e*(*u*) ≤ *d*_*ss*_(*u*,*v*) + *e*(*v*), since *e*(*v*) ≥ *d*_*ss*_(*v*,*x*). i.e. 0 ≤ *e*(*u*) - *e*(*v*) ≤ *d*_*ss*_(*u*,*v*).Therefore |*e*(*u*) - *e*(*v*)| ≤ *d*_*ss*_(*u*,*v*).

### **Remark3.11**.

Note that in crisp graph, for every two adjacent nodes *u* and *v*, |*d*(*u*,*x*) - *d*(*v*,*x*)| ≤ 1. In Figure 2, *u*_1_ and *u*_2_ are adjacent nodes. Note that *d*_*ss*_(*u*_1_,*u*_3_) = 3.65 and *d*_*ss*_(*u*_2_,*u*_3_) = 1.1 and hence |*d*_*ss*_(*u*_1_,*u*_3_)) - *d*_*ss*_(*u*_2_,*u*_3_)| = 2.55. But this result is true if *u* and *v* are strong neighbors as in following theorem.

### **Theorem3.12**.

For every two strong neighbors *u* and *v* in a connected fuzzy graph *G*:(*V*,*σ*,*μ*), |*d*_*ss*_(*u*,*x*) - *d*_*ss*_(*v*,*x*)| ≤ 1 for every node *x* of *G*.

### **Proof**.

Let *u* and *v* be strong neighbors in *G* and let *x* be any node of *G*. Assume *d*_*ss*_(*u*,*x*) ≥ *d*_*ss*_(*v*,*x*). Then by triangle inequality we have *d*_*ss*_(*u*,*x*) ≤ *d*_*ss*_(*u*,*v*) + *d*_*ss*_(*v*,*x*). Since *u* and *v* are strong neighbors *d*_*ss*_(*u*,*x*) ≤ 1 + *d*_*ss*_(*v*,*x*) ⇒ 0 ≤ *d*_*ss*_(*u*,*x*) - *d*_*ss*_(*v*,*x*) ≤ 1. Therefore |*d*_*ss*_(*u*,*x*) - *d*_*ss*_(*v*,*x*)| ≤ 1.

### **Proposition3.13**.

For any two real numbers *a*, *b* such that 0 <*a* ≤*b* ≤ 2*a*, there exist a fuzzy graph *G* such that *r*(*G*) = *a* and *d*(*G*) = *b*.

### **Proof**.

**Figure 3:**
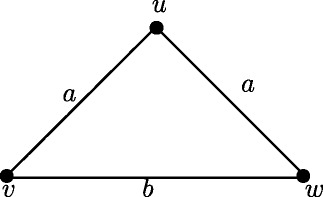
Fuzzy graph with all arcs strong.

From the definiton of strong arcs it follows that all arcs in Figure 3 are strong arcs. Therefore *d*_*ss*_(*u*,*v*) = *a*, *d*_*ss*_(*u*,*w*) = *a* and *d*_*ss*_(*v*,*w*) = *b*. Then *e*(*u*) = *a*, *e*(*v*) = *b* and *e*(*w*) = *b*. Therefore *r*(*G*) = *a* and *d*(*G*) = *b*.

### **Theorem3.14**.

If *G*:(*V*,*σ*,*μ*) is a self centered fuzzy graph, then each node of *G* is eccentric.

### **Proof**.

Assume *G* is self centered and let *u* be any node of *G*. Let *v* be an eccentric node of *u* i.e. *u*^∗^ = *v*. Then *e*(*u*) = *d*_*ss*_(*u*,*v*). Since *G* is self centered we have *e*(*v*) = *e*(*u*). Therefore *e*(*u*) = *d*_*ss*_(*u*,*v*) = *e*(*v*), which shows *u* is an eccentric node of *v* i.e. *v*^∗^ = *u*. Hence the proof.

### **Remark3.15**.

**Figure 4:**
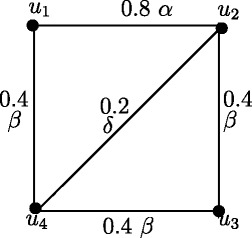
Self centered fuzzy graph.

Fuzzy graph *G*:(*V*,*σ*,*μ*) in Figure 4 is self centered with *e*(*u*_*i*_) = 0.8, *i* = 1,2,3,4. The condition in Theorem 3.14 is not sufficient. In Figure 1, each node is eccentric but *G* is not self centered.

### **Theorem3.16**.

If *G*:(*V*,*σ*,*μ*) is a self centered fuzzy graph, then for every pair of nodes *u*,*v* ∈*G*, *u* ∈*V*^∗^ implies *v* ∈*U*^∗^, where *U*^∗^ is the set of all eccentric nodes of *u* and *V*^∗^ is the set of all eccentric nodes of *v*.

### **Proof**.

Assume *G* is self centered and let *u*, *v* be any two nodes of *G*. Let *u* be an eccentric node of *v*. i.e. *d*_*ss*_(*v*,*u*) = *e*(*v*), so we have *u* ∈*V*^∗^. Now it is required to prove that *v* ∈*U*^∗^. Since *G* is self centered we have *e*(*v*) = *e*(*u*). Also we have *d*_*ss*_(*v*,*u*) = *d*_*ss*_(*u*,*v*) = *e*(*v*). Therefore *e*(*u*) = *d*_*ss*_(*u*,*v*) which shows *v* is an eccentric node of *u* i.e. *v* ∈*U*^∗^. Hence the proof.

### **Remark3.17**.

The condition in Theorem 3.16 is not sufficient. In Figure 1, each node is eccentric and we have *u*^∗^ = *v*, *v*^∗^ = *u* and *w*^∗^ = *x*, *x*^∗^ = *w* but *G* is not self centered.

### **Remark3.18**.

**Figure 5:**
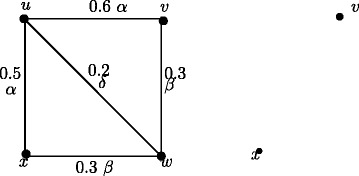
Fuzzy graph *G*:(*V*,*σ*,*μ*)<*C*(*G*)> with disconnected center.

The center of a connected fuzzy graph need be connected as shown in Figure 5.

### **Theorem3.19**.

The center of every connected fuzzy graph *G*:(*V*,*σ*,*μ*) lies in a block of *G*^∗^.

### **Proof**.

Let *G*:(*V*,*σ*,*μ*) be a connected fuzzy graph. Assume that the center of *G* does not lie in a block of *G*^∗^. Then there exist a node *v* such that, *v* is a cut node of *G*^∗^. Let *G*_1_ and *G*_2_ be any two components of *G*^∗^ - *v*. Therefore each of *G*_1_ and *G*_2_ contain at leat one central node of *G*. Let *u* be a node of *G* such that *d*_*ss*_(*u*,*v*) = *e*(*v*). Let *P*_1_ be a strong *u*−*v* path such that *d*_*ss*_(*u*,*v*) = *L*(*P*_1_), length of *P*_1_. Then one of *G*_1_ and *G*_2_ contains no node in the path *P*_1_, say *G*_2_ contains no node of *P*_1_. Let *w* be a central node of *G* that belongs to *G*_2_ and let *P*_2_ be a strong *v*−*w* path such that *d*_*ss*_(*v*,*w*) = *L*(*P*_2_), length of *P*_2_. Therefore *d*_*ss*_(*u*,*w*) = *L*(*P*_1_) + *L*(*P*_2_). Hence we have *e*(*w*) > *e*(*v*), which contradicts *w* is a central node of *G*. Hence center of every connected fuzzy graph *G*:(*V*,*σ*,*μ*) lies in a block of *G*^∗^.

### **Remark3.20**.

As in crisp graphs, in fuzzy graphs every peripheral node is an eccentric node but not conversly. In Figure 1, *u*,*v*,*w*,*x* are eccentric nodes but *w* and *x* are not peripheral nodes.

### **Remark3.21**.

In crsip graph no cutnode is a peripheral node but there are fuzzy graphs with peripheral nodes as fuzzy cut nodes. In Figure 1, nodes *u* and *v* are the peripheral nodes and node *v* is a fuzzy cut node. Note that removal of the node *v* reduces strength of connectedness between the nodes *w* and *x*.

### **Remark3.22**.

**Figure 6:**
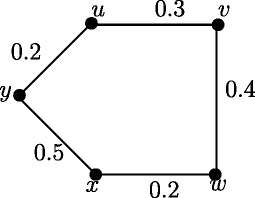
Fuzzy cycle which is not self centered.

A fuzzy cycle need not be self centered. In Figure 6, *r*(*G*) = 0.6 and *d*(*G*) = 0.7 and the central node is *v*.

## Self centered fuzzy cycle

Using the concept of *μ*−eccentric nodes, in (Sunitha and Vijayakumar 1998) Sunitha and Vijayakumar has proved the sufficient conditions for a fuzzy graph *G* such that *G*^∗^ is a cycle to be self centered. In this section, sufficient conditions for a cycle to be self centered based on strong sum distance are discussed. Note that all arcs in a fuzzy cycle are strong (Rosenfeld 1975).

### **Theorem4.1**.

Let *G*:(*V*,*σ*,*μ*) be a fuzzy graph with *n* nodes such that *G*^∗^ ≅ *C*_*n*_, cycle on *n* nodes with arcs *e*_*i*_ = (*u*_*i*_,*u*_*i*+1_) *i*=1,2,⋯,*n*−1 and *e*_*n*_ = (*u*_*n*_,*u*_1_). Let 0<*t*<*s*≤1. Then *G* is self centered if

1. *μ*(*e*_*i*_) = *t* for *i*=1,3,5,⋯,*n*−1, *μ*(*e*_*i*_) = *s* for *i*=2,4,6,⋯,*n*−2 and *μ*(*e*_*n*_) = *s* when *n* is even.
2. *μ*(*e*_*i*_) = *s* for *i*=1,3,5,⋯,*n*−2, *μ*(*e*_*i*_) = *t* for *i*=2,4,6,⋯,*n*−1 and *μ*(*e*_*n*_) = *s* when *n* is odd and *n*=4*k*−1, where *k*=1,2,3,⋯.
3. *μ*(*e*_*i*_) = *t* for *i*=1,3,5,⋯,*n*−2, *μ*(*e*_*i*_) = *s* for *i*=2,4,6,⋯,*n*−1 and *μ*(*e*_*n*_) = *t* when *n* is odd and *n*=4*k*+1, where *k*=1,2,3,⋯.

Also,

$${}</p><p class="noindent">r(G) = \left\{ \begin{array}{rl} & k(t+s), n = 4k \textit{ or } n = 4k + 1, k = 1,2,3, \cdots\\ & k(t+s) - t, n = 4k - 1, k = 1,2,3, \cdots\\ & k(t+s) + t, n = 4k + 2, k = 1,2,3, \cdots \end{array} \right. $$

### **Illustration1**.

Take *t* = 0.3 and *s* = 0.4. Figures 7, 8, 9, 10, 11, 12, 13 and 14 illustrates the above theorem.

**Figure 7:**
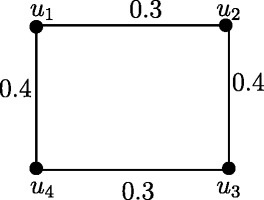
Fuzzy Cycle *C*_4_.

**Figure 8:**
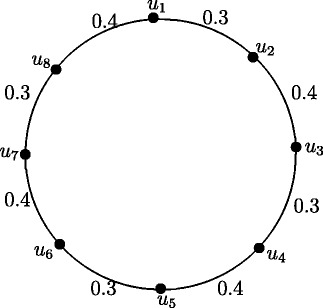
Fuzzy Cycle *C*_8_.

**Figure 9:**
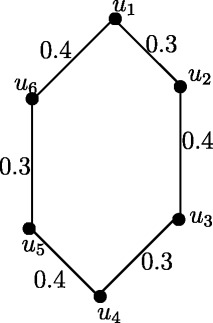
Fuzzy Cycle *C*_6_.

**Figure 10:**
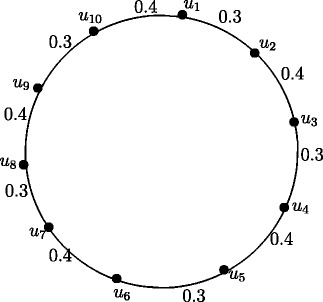
Fuzzy Cycle *C*_10_.

**Figure 11:**
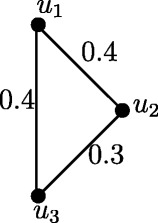
Fuzzy Cycle *C*_3_.

**Figure 12:**
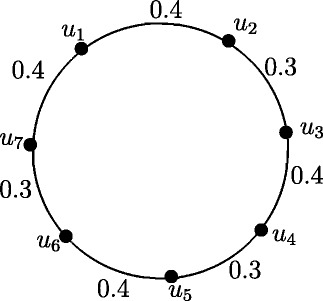
Fuzzy Cycle *C*_7_.

**Figure 13:**
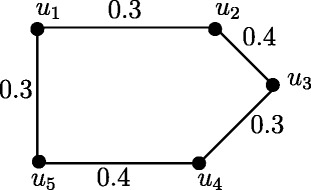
Fuzzy Cycle *C*_5_.

**Figure 14:**
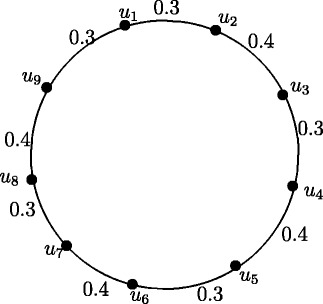
Fuzzy Cycle *C*_9_.

Case 1. *n* is even and *n*=4*k* where *k* = 1,2, *r*(*C*_4_) = 0.7 and *r*(*C*_8_) = 1.4.

Case 2. *n* is even and *n*=4*k*+2 where *k* = 1,2, *r*(*C*_6_) = 1.0 and *r*(*C*_10_) = 1.7.

Case 3. *n* is odd and *n*=4*k*−1 where *k* = 1,2, *r*(*C*_3_) = 0.4 and *r*(*C*_7_) = 1.1.

Case 4. *n* is odd and *n*=4*k*+1 where *k* = 1,2, *r*(*C*_5_) = 0.7 and *r*(*C*_9_) = 1.4.

## Strong sum distance in complete fuzzy graph

In (Mini Tom Sunitha 2014) Mini and Sunitha proved that any *u*−*v* path *P* in a CFG is a strongest path if and only if either *u* or *v* is a weakest node in the path. In this section we first prove a necessary and sufficient condition for all paths in a CFG to be strongest and then a necessary and sufficient condition for a CFG to be self centered. Note that in a CFG all arcs are strong (Rosenfeld 1975; Bhutani and Rosenfeld 2003a).

### **Remark5.1**.

**Figure 15:**
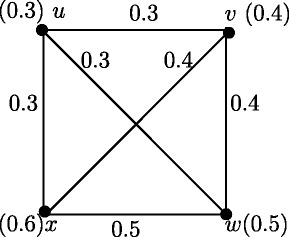
Complete Fuzzy graph which is not self centered.

A complete fuzzy graph need not be self centered. In Figure 15, *r*(*G*) = 0.3 and *d*(*G*) = 0.5 and the central node is *u*.

### **Theorem5.2**.

Let *G*:(*V*,*σ*,*μ*) be a CFG with *σ*^∗^ = {*u*_1_,*u*_2_,*u*_3_,...,*u*_*n*_} such that *σ*(*u*_1_) ≤ *σ*(*u*_2_) ≤ *σ*(*u*_3_) ≤ ⋯⋯ ≤ *σ*(*u*_*n*_). Then the strong sum distance between any two nodes *u*_*i*_,*u*_*j*_ in *G* is either *μ*(*u*_*i*_,*u*_*j*_) or 2 *σ*(*u*_1_).

### **Proof**.

Let *u*_*i*_,*u*_*j*_ be any two nodes in *G*. We have *d*_*ss*_(*u*_*i*_,*u*_*j*_) = min {*μ*(*u*_*i*_,*u*_*j*_), *μ*(*u*_*i*_,*u*_*k*_) + *μ*(*u*_*k*_,*u*_*j*_)}. Since *G* is CFG we have *μ*(*u*_*i*_,*u*_*k*_) = *σ*(*u*_*i*_) ∧ *σ*(*u*_*k*_). Also since *σ*(*u*_1_) ≤ *σ*(*u*_*i*_) for *i* = 2,3, ⋯, *n*, when *k* = 1, *μ*(*u*_*i*_,*u*_1_) = *σ*(*u*_1_) and *μ*(*u*_1_,*u*_*j*_) = *σ*(*u*_1_). Therefore *d*_*ss*_(*u*_*i*_,*u*_*j*_) = min {*μ*(*u*_*i*_,*u*_*j*_), 2 *σ*(*u*_1_)}.

### **Theorem5.3**.

Let *G*:(*V*,*σ*,*μ*) be a complete fuzzy graph on *n* nodes, n ≥3. All paths in *G* are strongest paths if and only if there is at most one node *w* in *G* having different node strength and *σ*(*w*) > *σ*(*u*_*i*_)*i* = 1,2,3, ⋯,*n*−1.

### **Proof**.

Let *G*:(*V*,*σ*,*μ*) be a complete fuzzy graph on *n* nodes, n ≥3, with *σ*^∗^ = {*u*_1_,*u*_2_,*u*_3_,...,*u*_*n*−2_,*v*,*w*} and all nodes *u*_*i*_, *i*=1,2,3...,*n*−2 have same node strength. Assume all paths in *G* are strongest paths. Suppose *σ*(*v*) ≠ *σ*(*w*) ≠ *σ*(*u*_*i*_), *i*=1,2,3,⋯,*n*−2. Case 1 : *σ* (*u*_*i*_) < *σ* (*w*) and *σ* (*u*_*i*_) < *σ* (*v*), *i*=1,2,3,⋯,*n*−2.Let *P* : *w*−*u*_1_−*u*_2_−.......−*u*_*k*_−*v*, *k* ≤ *n*−2, be a *w*−*v* path. Then *P* is not a strongest *w*−*v* path since neither *w* nor *v* is a weakest node in *P* (Mini Tom Sunitha 2014), contradiction.Case 2 : *σ* (*u*_*i*_) > *σ* (*w*) and *σ* (*u*_*i*_) > *σ* (*v*), *i*=1,2,3,⋯,*n*−2.Let *P* be any *u*_*i*_−*u*_*j*_ path, *i*,*j* = 1,2,3, ⋯,*n*−2, *i*≠*j* with either *v* or *w* as an internal node. Then *P* is not a strongest *u*_*i*_−*u*_*j*_ path since neither *u*_*i*_ nor *u*_*j*_ is a weakest node in *P* (Mini Tom Sunitha 2014), contradiction.Case 3 : *σ* (*u*_*i*_) < *σ* (*w*) and *σ* (*u*_*i*_) > *σ*(*v*), *i*=1,2,3,⋯,*n*−2.Let *P* be any *u*_*i*_−*w* path, *i* = 1,2,3, ⋯,*n*−2, with *v* as an internal node. Then *P* is not a strongest *u*_*i*_−*w* path since neither *u*_*i*_ nor *w* is a weakest node in *P* (Mini Tom Sunitha 2014), contradiction.Hence there exist at most one node *w* in *G* having different node strength. Next to prove *σ*(*w*) > *σ*(*u*_*i*_)*i* = 1,2,3, ⋯,*n*−1. Suppose not let, *σ*(*w*) < *σ*(*u*_*i*_)*i* = 1,2,3, ⋯,*n*−1.Then by case 2, we arrive at a contradiction. Hence *σ*(*w*) > *σ*(*u*_*i*_)*i* = 1,2,3, ⋯,*n*−1.

Conversely assume that there is at most one node *w* in *G* having different node strength and *σ*(*w*) > *σ*(*u*_*i*_)*i* = 1,2,3, ⋯,*n*−1. Then any path *P*, joining any two nodes in *G* is such that at least one of the end nodes of *P* is a weakest node in the path *P* and hence *P* is a strongest path (Mini Tom Sunitha 2014).

### **Theorem5.4**.

Let *G*:(*V*,*σ*,*μ*) be a CFG on *n* nodes, n ≥3. Then *G* is self centered if and only if all paths in *G* are strongest paths.

### **Proof**.

Let *G*:(*V*,*σ*,*μ*) be a CFG. Assume *G* is self centered. Then by Theorem 3.14, each node of *G* is eccentric. Also for any two nodes *u*,*v* in *G*, *e*(*u*) = *e*(*v*) = *r*(*G*) = *d*(*G*). If possible assume that all paths in *G* are not strongest paths. Therefore by Theorem 5.3, there exist at least two nodes *u*,*v* with different node strength and let *w* be an arbitrary node in *G* such that *σ*(*w*) is least. i.e. we have *σ*(*w*) < *σ*(*u*) and *σ*(*w*) < *σ*(*v*). Also we have *μ*(*u*,*v*) = *σ*(*u*) ∧*σ*(*v*) > *σ*(*w*) and *d*_*ss*_(*u*,*v*) = Min {*μ*(*u*,*v*), 2 *σ*(*w*) } by Theorem 5.2. Therefore *d*_*ss*_(*u*,*v*) > *σ*(*w*). Also we have *e*(*u*) = max {*d*_*ss*(_*u*,*v*):*v* ∈*V*}.

Therefore *e*(*u*) > *σ*(*w*) ⋯⋯⋯(1).

Now, for any node *u* in *G* we have *μ*(*u*,*w*) = *σ*(*w*) and therefore *d*_*ss*_(*u*,*w*) = *σ*(*w*) by Theorem 5.2.

Thus *e*(*w*) = max {*d*_*ss*_(*w*,*u*):*u* ∈*V*} = *σ*(*w*) ⋯⋯⋯(2).

From (1) and (2) *e*(*u*) > *e*(*w*), which contradicts our assumption that *G* is self centered. Hence all paths in *G* are strongest paths.

Conversely assume all paths in *G* are strongest paths. Since all paths in *G* are strongest paths, there is at most one node in *G* having different strength and the strength of such a node is greater than the strength of all other nodes in *G* by Theorem 5.3. Hence all arcs in *G* have same strength. Also *d*_*ss*_(*u*,*v*) = *μ*(*u*,*v*) ∀*u*,*v* by Theorem 5.2. Hence for any two node *u*,*v* in *G*, *e*(*u*) = *e*(*v*). Therefore *G* is self centered.

## Embedding theorem

In this section, we shall consider the construction of a fuzzy graph *G* from a fuzzy graph *H* such that <*C*(*G*)> ≅*H*.

### **Theorem6.1**.

Let *H*:(*V*,*σ*^′^, *μ*^′^) be a fuzzy graph. Then there exists a connected fuzzy graph *G*:(*V*,*σ*,*μ*) such that <*C*(*G*)> ≅*H*.

### **Proof**.

Let 0 <*c* = ∧*σ*^′^(*u*). Construct a fuzzy graph *G*:(*V*,*σ*,*μ*) from *H* as follows. Take four new nodes *u*_1_,*u*_2_,*v*_1_,*v*_2_ and put *σ*^∗^ = *σ*^′∗^ ∪ { *u*_1_, *u*_2_, *v*_1_, *v*_2_ } where *σ* = *σ*^′^ for all nodes *w* in *H*, *μ* = *μ*^′^ for all arcs (*u*,*v*) in *H*. Let *σ*(*u*_*i*_) = *σ*(*v*_*i*_) = *t* (*t* ≤ *c*),*i* = 1,2; *μ*(*u*_1_,*u*_2_) = *μ*(*v*_1_,*v*_2_) = *t* and *μ*(*u*_2_,*w*) = *μ*(*v*_1_,*w*) = *t* ∀*w* ∈*H*. Then clearly *G*:(*V*,*σ*,*μ*) is a fuzzy graph and by definition of strong arcs, the arcs (*u*_1_,*u*_2_), (*v*_1_,*v*_2_), (*u*_1_,*w*), (*v*_1_,*w*) ∀*w* ∈*H* are strong arcs. Also we have *e*(*w*) = 2*t* ∀*w* ∈*H* and *e*(*u*_1_) = *e*(*v*_1_) = 3*t* and *e*(*u*_2_) = *e*(*v*_2_) = 4*t*. Thus <*C*(*G*)> ≅*H* and *r*(*G*) = 2*t* and *d*(*G*) = 4*t*.

### **Example6.2**.

**Figure 16:**
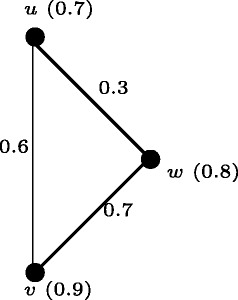
Fuzzy Graph *H*:(*V* ^′^,*σ* ^′^,*μ* ^′^).

**Figure 17:**
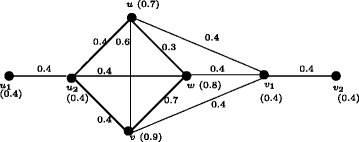
Fuzzy Graph *G*:(*V*,*σ*,*μ*) where Center <*C*(*G*)>≅*H*.

Let *H*:(*V*^′^,*σ*^′^, *μ*^′^) be a fuzzy graph (Figure 16) with *V*^′^={*u*,*v*,*w*}. Let *σ*^′^(*u*)=0.7, *σ*^′^(*v*)=0.9, *σ*^′^(*w*)=0.8, *μ*^′^(*u*,*v*)=0.6, *μ*^′^(*v*,*w*)=0.7 and *μ*^′^(*u*,*w*)=0.3. Construct the fuzzy graph *G*:(*V*,*σ*,*μ*) (Figure 17) from *H* as follows. Take *t* = 0.4. The node set of *G* is *V*=*V*^′^ ∪ { *u*_1_, *u*_2_, *v*_1_, *v*_2_ }. Let *σ*(*u*)=0.7, *σ*(*v*)=0.9, *σ*(*w*)=0.8, *σ*(*u*_1_)=*σ*(*u*_2_)=*σ*(*v*_1_)=*σ*(*v*_2_)=0.4, *μ*(*u*,*v*)=0.6, *μ*(*v*,*w*)=0.7, *μ*(*u*,*w*)=0.3, *μ*(*u*_1_,*u*_2_)=0.4, *μ*(*v*_1_,*v*_2_)=0.4, *μ*(*u*_2_,*u*)=0.4, *μ*(*u*_2_,*v*)=0.4, *μ*(*u*_2_,*w*)=0.4, *μ*(*v*_1_,*u*)=0.4, *μ*(*v*_1_,*v*)=0.4, *μ*(*v*_1_,*w*)=0.4. Note that *e*(*u*)=*e*(*v*)=*e*(*w*)=0.8 and *e*(*u*_2_)=*e*(*v*_1_)=1.2 and *e*(*u*_1_)=*e*(*v*_2_)=1.6. Thus <*C*(*G*)> ≅*H* and *r*(*G*) = 0.8 and *d*(*G*) = 1.6.

## Strong sum distance in fuzzy trees

Many metric properties of fuzzy trees are studied using *μ*− distance (Sunitha and Vijayakumar 1998), *g*− distance (Sameena K and Sunitha 2011) and fuzzy detour *g*− distance (Linda and Sunitha 2014a). In this section a similar study is carried out on fuzzy trees using strong sum distance. Note that an f-graph *G*:(*V*,*σ*,*μ*) is an f-tree if and only if it has no *β* strong arcs (Sunil and Sunitha 2009).

### **Theorem7.1**.

Let *G*:(*V*,*σ*,*μ*) be a fuzzy tree and *F*:(*V*,*σ*,*ν*) be the maximum spanning tree of *G*. Then for each node *u* in *G*, *e*(*u*) in *G* is same as *e*(*u*) in *F*.

### **Proof**.

Let *G*:(*V*,*σ*,*μ*) be a fuzzy tree and let *u* be any arbitrary node in *G*. Let *e*(*u*) = *k*, i.e. ∃ a node *v* in *G* such that *d*_*ss*_(*u*,*v*) = *k*, which implies that there is strong *u*−*v* path *P* in *G* such that *L*(*P*) = *k*. Since *G* is an f-tree, *P* is the unique strong *u*−*v* path in *G*. Let *F*:(*V*,*σ*,*ν*) be the maximum spanning tree of *G*. Since *G* is a fuzzy tree, *F* is the unique maximum spanning tree of *G* and contains all strong arcs of *G*. Thus *F* contains the unique strong *u*−*v* path and *d*_*ss*_(*u*,*v*) = *k*. Hence *e*(*u*) = *k* in *F*.

### **Corollary7.2**.

Let *G*:(*V*,*σ*,*μ*) be a fuzzy tree and *F*:(*V*,*σ*,*ν*) be the maximum spanning tree of *G*. Then <*C*(*G*)> ≅ <*C*(*F*)> and center of a fuzzy tree consists of either one or two neighboring nodes.

### **Remark7.3**.

**Figure 18:**
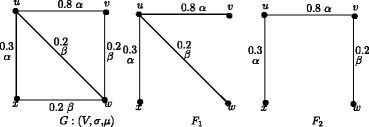
A fuzzy graph *G* which is not an f-tree and its MST’s *F*_1_ and *F*_2_.

Theorem 7.1 does not hold for a fuzzy graph which is not a fuzzy tree. The fuzzy graph *G* in Figure 18 is not a fuzzy tree and hence *G* has more than one maximum spanning tree. We have *e*(*u*) = 0.4, *e*(*v*) = 0.4, *e*(*w*) = 0.2 and *e*(*x*) = 0.4 in *G*, *e*(*u*) = 0.8, *e*(*v*) = 1.1, *e*(*w*) = 0.5 and *e*(*x*) = 1.1 in *F*_1_ and *e*(*u*) = 1, *e*(*v*) = 1.1, *e*(*w*) = 1.3 and *e*(*x*) = 1.3 in *F*_2_. Note that the eccentricity of node *u* is not same in *G*, *F*_1_ and *F*_2_.

### **Theorem7.4**.

Let *G*:(*V*,*σ*,*μ*) be a fuzzy tree and *F*:(*V*,*σ*,*ν*) be the maximum spanning tree of *G*. Then *G* and *F* have the same set of eccentric nodes.

### **Proof**.

Let *G*:(*V*,*σ*,*μ*) be a fuzzy tree and *F*:(*V*,*σ*,*ν*) be the maximum spanning tree of *G*. By Theorem 7.1, for any node *u*, eccentricity of *u* in *G* is same as eccentricity of *u* in *F*. Let *e*(*u*) = *k* and let *u*^∗^ = *v* in *G*. Then there is a strong *u*−*v* path *P* in *G*, which is also in *F* such that *L*(*P*) = *k*. Therefore *u*^∗^ = *v* in *F* also. Similarly we can prove that for any node *u* in *F*, if *u*^∗^ = *v* in *F* then *u*^∗^ = *v* in *G* also. Hence the proof.

### **Theorem7.5**.

An eccentric node of a fuzzy tree *G*:(*V*,*σ*,*μ*) is a fuzzy end node of *G*.

### **Proof**.

Let *G*:(*V*,*σ*,*μ*) be a fuzzy tree and let *u* be an eccentric node of *G*. Then *u* is an eccentric node of *F*, the maximum spanning tree of *G* by Theorem 7.1. Since *F* is a tree *u* is an end node of *F* and hence a fuzzy end node of *F*. Since *G* and *F* have the same set of fuzzy end nodes (Sameena K and Sunitha 2011), *u* is fuzzy end node of *G*.

### **Remark7.6**.

**Figure 19:**
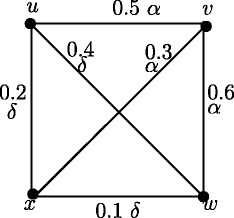
Fuzzy graph with fuzzy end node which is not eccentric node.

The converse of Theorem 7.5 does not hold as we see in Figure 19. In Figure 19, *x* is a fuzzy end node. We have *u*^∗^ = *w*, *v*^∗^ = *w*, *w*^∗^ = *u* and *x*^∗^ = *w*. Thus *x* is not an eccentric node of *G*.

### **Remark7.7**.

Theorem 7.5 does not hold for a fuzzy graph which is not a fuzzy tree as we see in Figure 18. In Figure 18, the eccentric nodes of *G* are *u*,*v* and *x*, but *G* has no fuzzy end nodes.

### **Theorem7.8**.

Let *G*:(*V*,*σ*,*μ*) be a fuzzy tree. Then a node *u* of *G* is an eccentric node if and only if *u* is peripheral node of *G*.

### **Proof**.

Let *G*:(*V*,*σ*,*μ*) be a fuzzy tree and *u*, an eccentric node of *G*. Then by Theorem 7.5, *u* is fuzzy end node of *G*. Since *G* and *F* have same set of fuzzy end nodes (Sameena K and Sunitha 2011), *u* is fuzzy end node of *F*. Thus *u* is an end node of *F*. Choose a fuzzy end node *v* of *F* other than *u* such that *d*_*ss*_(*u*,*v*) in *F* is maximum. Note that such a node *v* exists since Theorem 2 of (Bhutani and Rosenfeld 2003c) sates that every fuzzy tree has at least two fuzzy end nodes. Then it follows that *d*_*ss*_(*u*,*v*) is the diameter of *F* and hence the diameter of *G*. Therefore *u* and *v* are peripheral nodes of *G*.

Converse part follows from Remark 3.20.

### **Remark7.9**.

From Theorem 7.5 and 7.8 we have the following Theorem.

### **Theorem7.10**.

A peripheral node of a fuzzy tree *G*:(*V*,*σ*,*μ*) is a fuzzy end node of *G*.

### **Remark7.11**.

**Figure 20:**
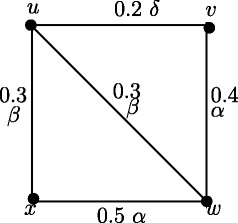
Fuzzy graph with peripheral node which is not fuzzy end node.

The converse of Theorem 7.10 is not true (Remark 7.6). Also note that Theorem 7.10 is true only in a fuzzy tree. In Figure 18, *u*, *v* and *x* are peripheral nodes, but *G* has no fuzzy end nodes. Also in Figure 20, *v* and *x* are peripheral nodes of *G*, but *x* is not a fuzzy end node of *G*.

## Boundary node of a fuzzy graph

Gary Chartrand and Ping Zang (2006) introduced the concepts of boundary vertex and boundary based on geodesic distance in crisp graph. Linda and Sunitha (2012) introduced the concepts of boundary node and boundary based on geodesic distance in fuzzy graphs. In this section we introduce the same concepts based on strong sum distance in fuzzy graphs. Also we define complete node based on strong sum distance and show by an example that a complete node need not be a boundary node.

### **Definition8.1**.

A node *v* in a connected fuzzy graph *G*:(*V*,*σ*,*μ*) is a boundary node of a node *u* if *d*_*ss*_(*u*,*v*) ≥ *d*_*ss*_(*u*,*w*) for each neighbor *w* of *v*; while a node *v* is a boundary node of a fuzzy graph *G* if *v* is a boundary node of some node of *G*.

The set of all boundary nodes of *u* is denoted by *u*^*b*^. The fuzzy subgraph induced by the boundary nodes of *G* is called the boundary of *G* denoted by *∂*(*G*).

### **Example8.2**.

**Figure 21:**
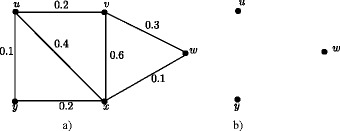
Boundary nodes in fuzzy Graph *G* based on strong sum distance.**(a)**. Boundary nodes **(b)**. Boundary *∂*(*G*).

Consider the fuzzy graph in Figure 21(a). Here *u*^*b*^ = {*w*,*y*}, *v*^*b*^ = {*u*}, *w*^*b*^ = {*u*}, *x*^*b*^ = {*w*}, *y*^*b*^ = {*w*}.

Hence the boundary nodes of *G* are *u*, *w* and *y*.

### **Remark8.3**.

In a connected fuzzy graph every eccentric node is a boundary node, but a boundary node need not be an eccentric node. Consider the fuzzy graph in Figure 21. The eccentric nodes *u* and *w* are boundary nodes of *G* but the boundary node *y* is not an eccentric node of *G*.

### **Example8.4**.

**Figure 22:**
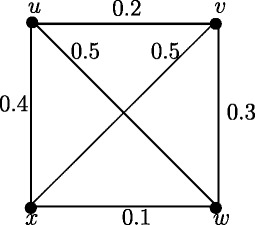
*G*:(*V*,*σ*,*μ*) which is not self centered.

In Figure 22, *e*(*u*) = 0.9 and *u*^∗^ = *v*, *e*(*v*) = 1.4 and *v*^∗^ = *w*, *e*(*w*) = 1.4 and *w*^∗^ = *v*, *e*(*x*) = 0.9 and *x*^∗^ = *w*. Also *u*^*b*^ = *v*, *v*^*b*^ = *w*, *w*^*b*^ = *v*, *x*^*b*^ = *w*. Hence the nodes *v*,*w* are peripheral nodes, eccentric nodes and boundary nodes. Note that *G* is not self centered.

### **Definition8.5**.

A node *v* in a fuzzy graph *G* is called a complete node if the fuzzy subgraph induced by the neighbors of *v* form a complete fuzzy graph.

### **Remark8.6**.

**Figure 23:**
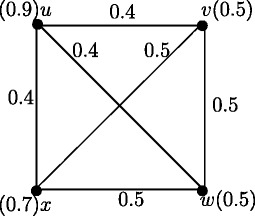
Complete node in a fuzzy graph.

In crisp graph a vertex *v* of *G* is a boundary vertex of every vertex distinct from *v* if and only if *v* is a complete vertex of *G* (Chartrand and Zang 2006) but in fuzzy graphs, based on strong sum distance a complete node need not be a boundary node as shown in Figure 23. Node *u* is a complete node but it is not a boundary node. Also it may be noted that a node which is a boundary node of all other nodes need not be complete. Node *w* is boundary node of all other nodes, but it is not complete.

### **Remark8.7**.

**Figure 24:**
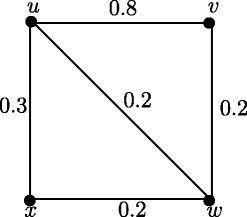
Fuzzy graph in which fuzzy cut node is a boundary node.

In crisp graphs no cut node of a connected graph *G* is a boundary node of *G* (Chartrand and Zang 2006), but in fuzzy graphs which is not a fuzzy tree, a fuzzy cut node can be a boundary node as in Figure 24. In Figure 24, the boundary nodes of *G* are *u*,*v* and *x*. Note that node *u* is a fuzzy cut node. Node *w* is neither fuzzy cut node nor boundary node.

### **Theorem8.8**.

In a fuzzy graph *G*:(*V*,*σ*,*μ*) a node *u* is cut node implies *u* is not a boundary node.

### **Proof**.

Let *G* be a fuzzy graph. Assume, to the contrary that there exists a cut node *u* of *G* such that *u* is a boundary node of some node *v* of *G*. Let *G*_1_ be the component of *G*−*u* which contains *v* and *G*_2_ be any other component of *G*−*u*. If node *w* is a neighbor of *u* that belongs to *G*_2_, then *d*_*ss*_(*v*,*w*) = *d*_*ss*_(*v*,*u*) + *x*, 0<*x*≤1, which contradicts our assumption that *u* is a boundary node of *v*.

### **Theorem8.9**.

In a fuzzy tree *G*:(*V*,*σ*,*μ*) no fuzzy cut node is a boundary node of *G*.

### **Proof**.

Let *G* be a fuzzy tree. Assume, to the contrary that there exists a fuzzy cut node *u* of *G* such that *u* is a boundary node of some node *v* of *G*. Let *F* be the unique maximum spanning tree of *G*. Theorem 8 of (Sunitha and Vijayakumar 1999) states that internal nodes of *F* are fuzzy cut nodes of *G*. Hence *u* is an internal node of *F*. By Theorem 7.4 and 7.5, *G* and *F* have the same set of eccentric nodes and an eccentric node of *G* is a fuzzy end node of *G*. Now since *u* is a boundary node it is an eccentric node of *G* and hence *u* is a fuzzy end node of *F*, which contradicts that *u* is an internal node of *F*. Hence no fuzzy cut node of a fuzzy tree is a boundary node.

### **Theorem8.10**.

In a fuzzy tree *G*:(*V*,*σ*,*μ*) a boundary node is a fuzzy end node.

### **Proof**.

Let *G* be a fuzzy tree and *u* is a boundary node of *G*. In a fuzzy tree every node is either a fuzzy cut node or a fuzzy end node (Bhutani and Rosenfeld 2003c). By Theorem 8.9 no fuzzy cut node is a boundary node of *G*. Hence *u* is a fuzzy end node of *G*.

### **Remark8.11**.

**Figure 25:**
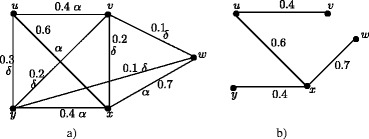
Fuzzy end nodes and boundary nodes in fuzzy graph *G* based on strong sum distance.**(a)**. Fuzzy end node is not a boundary node **(b)**. Unique maximum spanning tree.

In a fuzzy tree *G*:(*V*,*σ*,*μ*) a fuzzy end node need not be a boundary node. In Figure 25(a), the boundary nodes are *v* and *w* and the fuzzy end nodes are *v*, *w* and *y*. Note that the fuzzy end node *y* is not a boundary node.

### **Theorem8.12**.

Let *G*:(*V*,*σ*,*μ*) be a complete fuzzy graph on *n* nodes, *n*≥3 and let *u*_0_ be a node of *G*. Every node distinct from *u*_0_ is a boundary node of *u*_0_ if and only if *u*_0_ is a weakest node of *G*.

### **Proof**.

*n*≥3. Let *u*_0_ be a node of *G* such that every node distinct from *u*_0_ is a boundary node of *u*_0_. i.e; we have ${u_{0}^{b}}$ = {*u*_*i*_,*i*=1,2,3,...,*n*−1}. To prove that *u*_0_ is a weakest node of *G*. Suppose not. Let *u*_*k*_ be a node of *G* such that *σ*(*u*_*k*_) is least. Then by definition of CFG, *μ*(*u*_0_,*u*_*k*_) = *σ*(*u*_*k*_) and *μ*(*u*_0_,*u*_*i*_) = *σ*(*u*_0_) ∧*σ*(*u*_*i*_), *i*≠*k*. Therefore we have *μ*(*u*_0_,*u*_*k*_) < *μ*(*u*_0_,*u*_*i*_). Also, *d*_*ss*_(*u*_0_,*u*_*k*_) = *μ*(*u*_0_,*u*_*k*_) and *d*_*ss*_(*u*_0_,*u*_*i*_) = min {*μ*(*u*_0_,*u*_*i*_), 2 *σ*(*u*_*k*_)}, *i*≠*k* by Theorem 5.2. Clearly *d*_*ss*_(*u*_0_,*u*_*k*_) < *d*_*ss*_(*u*_0_,*u*_*i*_). Therefore by definition of boundary node *u*_*k*_ is not a boundary node of *u*_0_, which contradicts the assumption that every node distinct from *u*_0_ is a boundary node of *u*_0_. Therefore *u*_0_ is a weakest node of *G*.

Conversly assume that *u*_0_ is a weakest node of *G*. Then by definition of CFG, *μ*(*u*_0_,*u*_*i*_) = *σ*(*u*_0_), *i*=1,2,3,...,*n*−1. Also for any node *u*_*i*_ of *Gd*_*ss*_(*u*_0_,*u*_*i*_) = *μ*(*u*_0_,*u*_*i*_). Hence by definition of boundary node, every node distinct from *u*_0_ is a boundary node of *u*_0_.

### **Corollary8.13**.

In a CFG *G*:(*V*,*σ*,*μ*), if *u*_0_ is the unique weakest node then every node distinct from *u*_0_ are boundary nodes of *G*, whereas if the weakest node is not unique then all the nodes of *G* are boundary nodes of *G*.

## Interior node of a fuzzy graph

Gary Chartrand and Ping Zang (2006) introduced the concepts of interior vertex and interior based on geodesic distance in crisp graph. Linda and Sunitha (2012) introduced the concepts of interior node and interior based on geodesic distance in fuzzy graphs. In this section we introduce the same concepts based on strong sum distance in fuzzy graph. In crisp graph the interior nodes are precisely those nodes that are not boundary nodes (Chartrand and Zang 2006).

### **Definition9.1**.

Any node *w* in a connected fuzzy graph *G*:(*V*,*σ*,*μ*) is said to lie between two other nodes *u* and *v*(both different from *w*) with respect to strong sum distance if *d*_*ss*_(*u*,*v*) = *d*_*ss*_(*u*,*w*) + *d*_*ss*_(*w*,*v*).

### **Definition9.2**.

A node *w* is an interior node of a connected fuzzy graph *G*:(*V*,*σ*,*μ*) if for every node *u* distinct from *w*, there exist a node *v* such that *w* lies between *u* and *v*.

### **Example9.3**.

**Figure 26:**
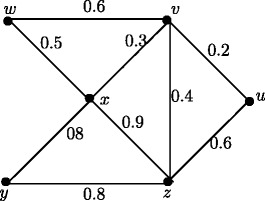
Fuzzy graph in which a node is neither an interior node nor a boundary node.

Consider the fuzzy graph in Figure 26. The interior nodes are *x*, *w* and *z* and boundary nodes are *u* and *v*. Node *y* is neither a boundary node nor an interior node.

### **Theorem9.4**.

Let *G*:(*V*,*σ*,*μ*) be a fuzzy graph. A boundary node of *G* is not an interior node of *G*.

### **Proof**.

Let *v* be a boundary node of a connected fuzzy graph *G* : (*V*,*σ*,*μ*), say *v* is a boundary node of the node *u*. Assume, to the contrary, that *v* is an interior node of *G*. Therefore by definition of interior node, there exist a node *w* distinct from *u* and *v* such that *v* lies between *u* and *w*. Let *P* be that strong *u*−*w* path in which *v* lies between *u* and *w*. i.e; *P* = *u*−*v*_1_−*v*_2_−*v*_3_−...−*v*=*v*_*j*_−*v*_*j*+1_−....−*v*_*k*_ = *w*, 1 <*j*<*k*. Now *v*_*j*+1_ ∈ *N*(*u*) and *d*_*ss*_(*u*,*v*_*j*+1_) = *d*_*ss*_(*u*,*v*) + *k*, 0<*k* ≤ 1, which contradicts that *v* is a boundary node of *u*.

### **Remark9.5**.

The converse of Theorem 9.4 is not always true. In fuzzy graph, all nodes which are not boundary nodes need not be interior nodes. In Figure 26, the boundary nodes are *u* and *v* and interior nodes are *x*,*w* and *z*. Node *y* is neither a boundary node nor an interior node.

### **Remark9.6**.

In a complete fuzzy graph *G* : (*V*,*σ*,*μ*), by Theorem 8.12 and Corollary 8.13 there exists at most node which is not a boundary node. Therefore in a CFG there exist at most one node which in an interior node. The interior node if it exists is the unique weakest node of *G*.

### **Example9.7**.

**Figure 27:**
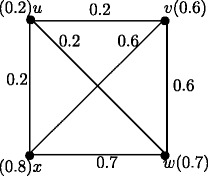
A complete fuzzy graph with an interior node.

In Figure 27, we have *d*_*ss*_(*u*,*v*) = 0.2, *d*_*ss*_(*u*,*w*) = 0.2, *d*_*ss*_(*u*,*x*) = 0.2, *d*_*ss*_(*v*,*w*) = 0.4, *d*_*ss*_(*v*,*x*) = 0.4, *d*_*ss*_(*w*,*x*) = 0.4 and *d*_*ss*_(*v*,*w*) = *d*_*ss*_(*v*,*u*) + *d*_*ss*_(*u*,*w*), *d*_*ss*_(*v*,*x*) = *d*_*ss*_(*v*,*u*) + *d*_*ss*_(*u*,*x*) and *d*_*ss*_(*w*,*x*) = *d*_*ss*_(*w*,*u*) + *d*_*ss*_(*u*,*x*). Hence *u* is the only interior node. By Theorem 8.12 nodes *v*,*w*,*x* are boundary nodes.

### **Theorem9.8**.

In a fuzzy tree *G*:(*V*,*σ*,*μ*) a node is an interior node if and only if it is a fuzzy cut node.

### **Proof**.

Let *G* be a fuzzy tree and *F*, the unique maximum spanning tree of *G*. Let *u* be an interior node of *G*. Hence by Theorem 9.4 *u* is not a boundary node of *G*. Suppose *u* is not a fuzzy cut node of *G*. Theorem 8 of (Sunitha and Vijayakumar 1999) states that internal nodes of *F* are fuzzy cut nodes of *G*. Hence *u* is not an internal node of *F*. *u* is an end node of *F* and hence a fuzzy end node of *G*. Thus *u* does not lie between any two nodes of *G* since there is only one strong arc incident on *u*. Therefore *u* is not an interior node of *G*, which contradicts our assumption. Hence *u* is a fuzzy cut node of *G*.

Conversely, let *u* be a fuzzy cut node of *G*. Hence *u* is an internal node of *F*. Since *F* is a tree, for any node *w* different *u* there exist an end node *v* of *F* such that *d*_*ss*_(*w*,*v*) = *d*_*ss*_(*w*,*u*) + *d*_*ss*_(*u*,*v*). Thus *u* is an interior node of *G*.

## Conclusion

The idea of strong sum distance which is a metric, in a fuzzy graph is introduced. The concepts of eccentricity, radius, diameter, center, self centered f-graphs etc. are studied using this metric. A characterization of self centered complete fuzzy graph is obtained and conditions under which a fuzzy cycle is self centered are established. A necessary and sufficient condition for all paths in a CFG with *n*≥ 3 to be strongest paths is obtained. Also discussed the construction of a fuzzy graph *G* from a given fuzzy graph *H* such that <*C*(*G*)> ≅*H*. We have proved that based on this metric, an eccentric node of a fuzzy tree *G* is a fuzzy end node of *G* and a node is an eccentric node of a fuzzy tree if and only if it is a peripheral node of *G* and the center of a fuzzy tree consists of either one or two neighboring nodes. The concepts of boundary nodes and interior nodes in a fuzzy graph based on strong sum distance are introduced. Some properties of boundary nodes, interior nodes and complete nodes are studied.
